# Medicalisation, suffering and control at the end of life: The interplay of deep continuous palliative sedation and assisted dying

**DOI:** 10.1177/1363459320976746

**Published:** 2020-12-11

**Authors:** Gitte Hanssen Koksvik, Naomi Richards, Sheri Mila Gerson, Lars Johan Materstvedt, David Clark

**Affiliations:** Norwegian University of Science and Technology, Norway; University of Glasgow, UK; University of Glasgow, UK; University of Glasgow, UK; Norwegian University of Science and Technology, Norway; University of Glasgow, UK

**Keywords:** assisted dying, control, good death, medicalisation, palliative sedation, suffering

## Abstract

Medicalisation is a pervasive feature of contemporary end of life and dying in Western Europe and North America. In this article, we focus on the relationship between two specific aspects of the medicalisation of dying: deep continuous palliative sedation until death and assisted dying. We draw upon a qualitative interview study with 29 health professionals from three jurisdictions where assisted dying is lawful: Flanders, Belgium; Oregon, USA; and Quebec, Canada. Our findings demonstrate that the relationship between palliative sedation and assisted dying is often perceived as fluid and complex. This is inconsistent with current laws as well as with ethical and clinical guidelines according to which the two are categorically distinct. The article contributes to the literature examining health professionals’ opinions and experiences. Moreover, our findings inform a discussion about emergent themes: suffering, timing, autonomy and control – which appear central in the wider discourse in which both palliative sedation and assisted dying are situated, and which in turn relate to the wider ideas about what constitutes a ‘good death’.

## Introduction

In Western Europe and North America, dying is increasingly preceded by clinical decision-making and most people die in medical institutions (Cohen et al., 2007; Rietjens et al., 2012). Medicine thereby plays a role in shaping what is considered a ‘good death’; a pain free, well-managed experience which also fulfils the ideals of individual freedom, control and adherence to personal preferences (Seymour et al., 2007; Walter, 1994). In this article, we address the highly medicalised end of life practice palliative sedation, sometimes called terminal sedation. Palliative sedation has been defined as

the intentional administration of sedative drugs in dosages and combinations required to reduce the consciousness of a terminal patient as much as necessary to adequately relieve one or more refractory symptoms (Broeckaert et al., 2002).

Palliative sedation is controversial and is generally considered an extreme measure to be undertaken only in prescribed circumstances (Materstvedt and Bosshard, 2009; Rietjens et al., 2018). Yet research evidence suggests that it occurs frequently (Abarshi et al., 2017; Scherrens et al., 2018). In Belgium, for example, which features in this study, a 2013 nationwide survey found that it was used in 12% of all deaths (Robijn et al., 2016). This notwithstanding, it is difficult to compare studies of the prevalence of sedation because different definitions of sedation are used by researchers (Arantzamendi et al., 2020; Kremling and Schildmann, 2020; Ten Have and Welie, 2014) and because palliative sedation and the medications employed is not necessarily documented.

Palliative sedation is sometimes proposed as an alternative to another controversial end of life practice: assisted dying (Seale et al., 2015; Seymour et al., 2007). Following international convention, we use assisted dying as an umbrella term for euthanasia, physician-assisted suicide and assisted suicide. By euthanasia we mean that a physician lawfully injects a lethal dose of medications to end a patient’s life, on the latter’s competent and voluntary request. In physician-assisted suicide, the patient lawfully receives medication from a physician for the purpose of ending their own life, whereas in assisted suicide, the medications may be supplied by a non-physician third party (Gerson et al., 2020). The palliative care field has traditionally staunchly opposed assisted dying and palliative care is defined by the World Health Organization as a practice which ‘intends neither to hasten or postpone death’ (WHO, 2020). For some, therefore, the inclusion of palliative sedation in palliative care practice illustrates that refractory symptoms and intolerable suffering can be countered without resorting to ending life (Scott, 2015: 145).

Assisted dying is currently available in a few jurisdictions worldwide, but it has become a significant part of cultural meaning-making surrounding death in many parts of the world (Richards and Krawczyk, 2019). Although palliative sedation and assisted dying are legally distinct, research shows that for both laypersons and clinicians the line between the two is not always clear-cut either conceptually or in practice (Bruinsma et al., 2014; Robjin et al., 2017; Seymour et al., 2015). Moreover, the presence or absence of lawful assisted dying has been found to influence care providers’ orientation towards palliative sedation, affecting the ways in which it is talked about, justified and carried out. In the Netherlands and Belgium, for example, Seale et al. (2015) found that care providers framed sedation as being an active decision and appropriate to bring an end to suffering which was perceived as pointless and when patients felt ‘unable to continue’. They contrast this to UK perceptions, a country where assisted dying is unlawful, where sedation is framed much more in terms of proportional symptom control and resorted to after much ‘trying’ and ‘struggling’ on the part of care providers (Seale et al., 2015).

In this article, we draw upon empirical data from a qualitative interview study conducted with 29 healthcare professionals involved with palliative care and assisted dying in Flanders, Belgium; Quebec, Canada; Oregon, USA. The overarching aim of our study was to investigate the relationship between assisted dying and palliative care in these three jurisdictions where assisted dying is lawful. In this article we focus specifically on palliative sedation, which is part of palliative care in all three jurisdictions. Our findings reveal a relationship between palliative sedation and assisted dying that is fluid and inconsistent both across and within jurisdictions. The two practices are conceived *either* as morally equivalent alternatives at end of life, different in degree only, or palliative sedation is seen as a lesser option to assisted dying. Our article contributes to the literature examining the opinions and experiences of health professionals. We discuss ways in which these findings elucidate various concerns with contemporary medicine and the issues affecting different cultures’ death systems. We identify some underlying themes: suffering, time, control and autonomy, and expand on the ways in which these figure into an understanding of what is considered a ‘good death’.

## Methods and conceptual clarifications

Most palliative care associations explicitly oppose assisted dying (Inbadas et al., 2017) and practitioners in the field who demur from this view are sometimes stifled, and fear being ostracised (BMJ, 2019). The reasons given for the opposition are the ethical principle of respect for life, that governments should prioritise investment in good palliative care, symptom control and social support which reduce requests for assisted dying, and that public demand for legalising assisted dying is fuelled by fundamental misconceptions about suffering at the end of life (Inbadas et al., 2017). Little empirical research exists which actually investigates ways in which palliative care practices interact with the implementation of assisted dying in different cultural and legal contexts (Gerson et al., 2020). The present study was conducted in Quebec, Flanders and Oregon, three jurisdictions where assisted dying is lawful. The objective was to explore the relationship between palliative care and assisted dying from the perspective of clinicians and other professionals involved in both assisted dying and palliative care. We wanted to go beyond official statements about anticipated or feared impacts of assisted dying legislation and learn about how it unfolds in practice. These jurisdictions were chosen as they have relatively comparable populations (4.1–8.4 million) within larger countries, however all have legalised different forms of assisted dying with differing eligibility criteria. In Oregon, adults who have mental capacity and are suffering from a terminal illness that is likely to produce death within no more than 6 months may qualify for physician-assisted suicide since 1997 (Oregon Death with Dignity Act 1997; see Oregon Health Authority, 2020). In Flanders, competent adults suffering intolerably from an irreversible somatic or psychiatric condition may apply for euthanasia or physician-assisted suicide, both legal since 2002 (Belgian Act on Euthanasia, 2002). In 2015, Québec legalised euthanasia for competent adults who suffer intolerably from an incurable physical condition at the end of life (Medical Aid in Dying 2015; see Gouvernement de Québec, 2020). Lastly, although their healthcare systems differ, each jurisdiction has advanced levels of palliative care development (Clark et al., 2019). Our study was exploratory in character, and we did not hypothesise any specific similarities or differences between the chosen locations.

We undertook purposive sampling to recruit professionals with a range of experiences working within palliative care and/or assisted dying in a setting where assisted dying is lawful. Participants were identified through extensive internet and literature searches, professional networks and snowballing. Twenty-nine professionals were interviewed (see Table 1).

**Table 1.** Participant information.

Participant ID^a^	Profession	Years of experience	Gender	Location	Recruitment
Professional 1	Academic	30+	M	Flanders	Direct contact
Physician 2	Physician	30+	M	Flanders	Direct contact
Physician 1	Physician	30+	M	Flanders	Snowball
Nurse 1	Nurse	20+	F	Flanders	Snowball
Physician 3	Physician	30+	M	Flanders	Snowball
Psych/sw/sp1	Spiritual care	20+	F	Flanders	Direct contact
Physician 4	Physician	30+	M	Flanders	Direct contact
Physician 5	Physician	20+	F	Flanders	Direct contact
Psych/sw/sp 2	Mental health	10+	M	Flanders	Snowball
Physician 6	Physician	20+	M	Flanders	Snowball
Professional 2	Administrator	30+	F	Oregon	Snowball
Professional 5	Social work, administrator	20+	F	Oregon	Direct contact
Professional 4	Nurse, administrator	20+	F	Oregon	Direct contact
Professional 3	Nurse, administrator	20+	F	Oregon	Direct contact
Nurse 2	Nurse	10+	F	Oregon	Snowball
Physician 7	Physician	30	M	Oregon	Snowball
Psych/sw/sp 3	Social work	10+	F	Oregon	Snowball
Physician 9	Physician	20+	M	Oregon	Snowball
Physician 8	Physician	20+	F	Oregon	Direct email
Physician 10	Physician	20+	M	Oregon	Snowball
Physician 12	Physician	30+	M	Quebec	Direct contact
Physician 14	Physician, administrator	30+	F	Quebec	Direct contact
Physician 13	Physician	30+	M	Quebec	Direct contact
Physician 17	Physician	30+	M	Quebec	Direct contact
Physician 15	Physician	20+	M	Quebec	Direct contact
Physician 16	Physician	10+	F	Quebec	Direct contact
Physician 11	Physician	30+	M	Quebec	Snowball
Physician 18	Physician	10+	F	Quebec	Snowball
Physician 19	Physician	40+	F	Quebec	Snowball

a Participant ID—Professional: any professional working in a non-clinical profession; Physician: includes all specialisms and areas of practice; Nurse: includes all specialisms and areas of practice; Psych/sw/sp: psychologists, social workers or spiritual counsellors.

The study obtained ethical clearance from the Research Ethics Committee of the University of Glasgow, College of Social Sciences (Application No. 400180010) and interviewees received detailed information about the study beforehand, providing written informed consent to participate. Participants were interviewed as individuals and not as representatives of their respective organisations, institutions or workplaces. Interviewees were asked the following: their experiences working either in palliative care with assisted dying or without; whether they had experienced or knew of differences in the field or practice of palliative care following the legalisation of assisted dying; their conception of the nature of the relationship between the two; their impressions of the general public’s knowledge and attitudes toward assisted dying and palliative care; and the challenges or benefits brought to palliative care by assisted dying. Authors 1 and 3 conducted the interviews (8 and 21, respectively): 24 took place face-to-face, 5 were conducted by telephone or Skype. Interviews lasted between 1 and 2 hours, and were audio recorded. Three were in French, the rest in English. All were transcribed verbatim and, in the case of those in French, translated by a professional agency.

Author 1 carried out a manual thematic coding of the interviews in NVivo12, using the major themes of the interview schedule as explained above as the basis for initial codes and engaging with the materials iteratively, carrying out the analysis in an inductive manner. New patterns and themes emerged through fine-grained reading. This yielded the practice of palliative sedation in relation to assisted dying as a recurring theme. For reliability, Author 3 conducted an independent thematic coding, similarly identifying palliative sedation as a theme. Interpretation of the findings were further deliberated within the writing team. All our interviewees’ recounting of practices and events are interpreted *as accounts*. Our interest is in how interviewees framed and construed practices and events and the different meanings given to them.

In the text, we wish to remain as true as possible to participants’ voices, favouring direct quotes. For example, interviewees from Oregon may refer to assisted dying as ‘Death with dignity’, which stems from the name of the law itself (Oregon Death with Dignity Act 1997; see Oregon Health Authority, 2020). Interviewees from Quebec say euthanasia or ‘MAiD’; shorthand for Medical Assistance in Dying, the name of the law specific to Quebec. In Belgium, euthanasia is the term most commonly used (Belgian Act on Euthanasia, 2002). We have altered some of the language to facilitate anonymization, corrected grammatical errors and removed repeated words. To further respect the anonymity of interviewees, we identify them only by profession and location, assigning a number to each.

## What is palliative sedation?

Palliative sedation is used to manage refractory symptoms; those that are particularly noxious and cannot be satisfactorily alleviated without lowering the patient’s consciousness. The degree of sedation varies from light to deep, and it can be used intermittently or maintained over time (Abarshi et al., 2017). Enck (1991) introduced the concept ‘terminal sedation’ to refer to deep and continuous sedation of patients at the end of life, which is maintained until death. Later, Materstvedt and Kaasa (2000) introduced the concept ‘palliative sedation’, which is currently the dominant term. However, the literature counts more than 50 definitions of this concept, covering various types and levels of sedation (Twycross, 2019). In the following we use the term ‘palliative sedation’ to designate only *deep sedation* where the patient is intentionally made fully unconscious – either gradually through an increase in medication over time or all at once – and where this level of unconsciousness is *maintained until death*.

Palliative sedation is initiated to alleviate uncontrollable suffering most commonly caused by delirium, dyspnoea or pain. Other symptoms include fatigue, nausea, agitation and existential distress such as profound feelings of meaninglessness, worthlessness, fear of death, loss of dignity and despair (Morita, 2004; Portenoy et al., 2015; Twycross, 2019).

Palliative sedation has been linked to assisted dying as some have argued that it may hasten or even cause death. This is despite evidence suggesting that it does not significantly shorten life (Beller et al., 2015; Maltoni et al., 2012). Ethicists and clinicians often discuss its possible life-shortening effects through the ‘principle of double effect’ (Anquinet et al., 2012; Boyle, 2004). In the philosophical literature the principle, which normally consists of four items that must be satisfied, comes in different formulations and the interpretation of each item is much discussed. Put briefly, the principle holds that an action that is morally good (in itself) is acceptable despite the possibility of it having bad consequences, provided the latter is only foreseen rather than intended, and on the condition that the action be proportionate. For palliative sedation that would mean: the action is morally good (in itself) because it alleviates extreme suffering, and therefore acceptable despite the possibility that it might also shorten survival time; still, that is not intended but merely foreseen. Additionally, the action is proportionate: dosages fit the symptoms.

In this vein, The European Association for Palliative Care outlines the differences between palliative sedation and euthanasia as follows:

In terminal sedation the *intention* is to relieve intolerable suffering, the *procedure* is to use a sedating drug for symptom control and the successful *outcome* is the alleviation of distress. In euthanasia the *intention* is to kill the patient, the *procedure* is to administer a lethal drug and the successful *outcome* is immediate death (Materstvedt et al., 2003).

## Ethical issues regarding palliative sedation and assisted dying

Despite theoretical attempts to separate the two practices, palliative sedation and assisted dying have been shown to be linked in practice. There is no research which systematically investigates either health professionals’ or the general public’s views on assisted dying in relation to palliative sedation. However, existing research displays systemic country variations in the conceptualisation and practice of palliative sedation (Seale et al., 2015; Seymour et al., 2015) and differences between the lay population’s conception of sedation and that of physicians (Morita et al., 2003).

Generally, the evidence suggests that the distinction between the two interventions is unclear in clinical practice both in countries where assisted dying is lawful and in countries where it is not (e.g. Anquinet et al., 2012; Papavasiliou et al., 2014; Robjin et al., 2017). For instance, Benítez-Rosario and Ascanio-León (2020) found that 80% of Spanish palliative care physicians were comfortable conducting palliative sedation for intractable physical symptoms, but that those who were not, considered palliative sedation to be a form of assisted dying in disguise. In a French study of an online citizen discussion forum about new end of life legislation, researchers found that palliative sedation was perceived as ‘a euthanasic practice’ or that it raised fears of a slippery slope leading to assisted dying (Toporski et al., 2017). In Belgium, Deyaert et al (2014) found that the concept of palliative sedation covers a range of practices in the minds of physicians who conduct them, identifying what the researchers call ‘primary’ or ‘secondary’ intentions to shorten life in over 40% of all reported cases. Others maintain a strictly consequentialist line of argument according to which the two are essentially the same (Juth et al., 2013). However, Magelssen et al (2016) have argued that focussing on physicians’ intentions may itself be misplaced, finding that this notion is largely foreign to physicians’ own deliberations.

Reports have shown increased rates of palliative sedation in several jurisdictions after, or coinciding with, assisted dying legislation (Commission Sur les Soins de Fin de Vie, 2019; Rietjens et al., 2008). Several studies indicate that families sometimes exert pressure on physicians to hasten death by increasing sedating medication disproportionately, in violation of the law and contrary to clinical guidelines (Anquinet et al., 2012; Seale et al., 2015). Evidence from the Netherlands indicates that physicians may actively encourage patients to opt for palliative sedation rather than assisted dying for reasons of bureaucracy, as the latter entails substantial paperwork (Bruinsma et al., 2013; Robjin et al., 2017). Both Anquinet et al. (2012) and Scherrens et al. (2018) report that informed of the two choices as alternatives, patients will have a preference for one or the other. Furthermore, palliative sedation is sometimes ritually organised similarly to assisted dying, including a family farewell (Bruinsma et al., 2014).

Having looked in some detail at palliative sedation as an end of life procedure and reviewing some of the most common ethical issues that surround it in relation to assisted dying, we now turn to our empirical findings.

## Perspectives from Quebec, Flanders and Oregon

### Assisted dying and palliative sedation as alternative medical interventions

For many interviewees, assisted dying and palliative sedation were perceived as alternatives that patients could opt for, depending on individual preferences:

In palliative sedation [you] live your life throughout your disease and you see where you can get, and for many patients you never come to the point that you need palliative sedation. (. . .) I think it will be always more frequent than euthanasia just because of that different mind-set, and it’s more the way most people want to live throughout their disease (Physician 1 Flanders).

Professionals viewed palliative sedation as a possible safety net for patients, allowing them to carry on, despite increasing symptom burden or fear thereof, in the knowledge that sedation would be an option should their condition become intolerable. Crucially, there is accordingly no need to pre-empt this decision or make plans. As one interviewee put it:

These are sort of two things in the same direction: one is the Death with Dignity [which] is more direct; you are titrating meds towards ending life. Whereas (. . .) in palliative sedation, we are titrating meds towards relief of symptoms. That’s a different goal than the Death with Dignity things (Physician 7 Oregon).

For others, the option of palliative sedation versus assisted dying was a choice between a sudden departure and a more gradual one:

Once we had a couple (. . .) and he wanted euthanasia and she didn’t want it [for him]. So, we had to go into a kind of discussion with her and her husband. We decided on doing palliative sedation, because that was acceptable for her because then she could let him go while he was sleeping. (. . .) And for him that was acceptable too, if you assured him that he wouldn’t be suffering or have pain. (. . .) That it could take him some days didn’t matter that much, but he really wanted to be asleep and not be there anymore awake (Physician 5 Flanders).

In this case, palliative sedation was a compromise. One indication from Oregon, where assisted dying is self-administered only and typically occurs away from any medical facility, is that some patients and their families ask if palliative sedation can be performed at home. This might suggest that some would prefer this ‘less confronting’ option. Other times, palliative sedation was considered appropriate in cases where a patient’s condition would preclude them from accessing assisted dying:

You push yourself further and further, and you push yourself to a point where euthanasia is simply impossible, and then for some people, well, it gets a bit tough, and you need that technique of palliative sedation (Physician 1 Flanders).

In cases of people who cannot have access to it, we certainly need to give the best care we can and to also suggest to people who really can’t take it anymore to go for palliative sedation, which is not exactly the same thing but it’s an option that works too (Physician 19 Quebec).

Other times, it seemed like physicians’ reticence to move forward with assisted dying created such situations:

We still have physicians who are ignoring the patients’ questions because they are not willing to do it (. . .) A lady (. . .) was sent in by the physician because she asked for euthanasia because she had so much pain. But they started to build up the Morphine and the Fentanyl and now she is so delirious that she couldn’t ask for euthanasia anymore - so now the discussion was if they could sedate her or not. That’s still happening (Physician 5 Flanders).

Here, palliative sedation arguably appears the less desirable of the two interventions. Some of our interviewees, however, believed that the lay public might wish for assisted dying due to a lack of knowledge about palliative care, available options, and because assisted dying is more frequently featured in the media. In their view, palliative care could cover what patients *really* wanted:

I always say ‘what [is] the reason you’re asking for this [assisted dying]? They tell me and then I ask them, ‘do you know what the other choices that you have are?’ They always say, ‘No, do I have other choices?’ (. . .) I explain all the other choices, and it happens frequently that a person changes their mind and tells me ‘Maybe I would prefer to just sleep a few days and then die, it’s going to be maybe more natural and my family is going to be more comfortable with this idea, and maybe me too’ (Physician 18 Quebec).

You realise that’s actually what you want: you don’t want to suffer. (. . .) I’ve heard that and some of my colleagues have told me they’ve heard it too, whereas people had first got here with the idea of physician-assisted dying on their minds (Physician 14 Quebec).

For these interviewees, what most people often wanted was not to suffer, yet appeared ill informed about options available within palliative care. As an Oregon interviewee explained however, there is a number of patients for whom a good death is about being in control.

Indeed, across the interviews, a consistent image emerged of the typical patient who requested assisted dying: ‘very private, very independent, very sure of themselves’ (Nurse 2 Oregon); people who ‘know what they want’ (Physician 10 Oregon); ‘who insist on having control on every element of their life’ and who would ‘rather die earlier’ than experience dependency (Physician 19 Quebec); and who ‘always want to control their life and so they want to control their death’ (Nurse 1 Flanders). Some recounted patients wanting assisted dying to exact revenge on their condition or reverse the stakes by ‘killing their disease’ before it could kill them. For these patients it would seem, the issue was one of leaving this world in control, a need which, it was proposed, hospice and palliative care ‘has a little more difficult time meeting’ (Physician 8 Oregon). Moreover, as an interviewee from Flanders observed: ‘One of the advantages of euthanasia is that it’s planned’ and ‘planning in healthcare is huge’ (Professional 1 Flanders). From this perspective once again, palliative sedation and assisted dying appear as complementary or alternative interventions responding to some extent to different conditions, but more importantly to different patients’ personalities and preferences.

### Assisted dying and palliative sedation: a matter of time

To some interviewees, there was no sharp distinction between palliative sedation and assisted dying. Indeed, sometimes the difference between the two was simply related to time:

In palliative care, we’re doing what we call palliative sedation, which is a procedure when we just give medication to the patient, so he sleeps until he dies (. . .) The assisted dying and the palliative sedation, it’s almost the same, the only difference is the delay in between the medication and the death, so for me, it’s the same thing (Physician 18 Quebec).

With euthanasia, you create that time-window for closure. That time window is guaranteed (. . .) There is a number of cases reported in [the] news in Belgium that were not terminally ill but they still created that time-window (. . .) that they filled in with closure of life (Professional 1 Flanders).

Several stories were relayed of interventions that had taken place prior to assisted dying legislation, where a physician was said to have performed euthanasia illegally. These acts were described as ‘more like continuous palliative sedation’, although possibly to excuse past illegal activity. Other times, palliative sedation was described in ways distinctly reminiscent of colloquial expressions for euthanasia; as ‘being put to sleep’ or ‘getting the injection’. Any substantive differences between the two were further questioned in reference to non-physical symptoms:

I think that especially when we started talking about terminal sedation for non-physical symptoms, then it was over. All patients who are tired of living, who no longer wish to live, who are sedated. . . Well, where’s the difference with asking for euthanasia? (Physician 17 Quebec).

The same interviewee described palliative sedation as a Trojan horse of assisted dying because ‘If you do this and it takes 48 hours, why don’t you do something that will last 5 minutes?’ This interviewee was strongly opposed to assisted dying. Nevertheless, several who were in favour of it expressed the same point:

I don’t know that it’s any more human to have somebody on terminal sedation for 20 hours where they have agonal breathing or are (. . .) essentially comatose through their medications and expire, rather than choosing to take medication and dying more quickly (Physician 9 Oregon).

The issue, for some, seems to be: if death can come quickly, what is the value of waiting?

### Palliative sedation as a lesser option to assisted dying

In Oregon, palliative sedation sometimes followed failed attempts at assisted dying when the patient ingested the prescribed lethal medications but did not die and was left in a liminal state. Palliative sedation then, by definition, became a lesser solution to an unfortunate situation:

I’m aware of several [failed attempts] that have happened where these folks then present to the hospice house for terminal care (. . .) And then basically they get terminal sedation (Physician 9 Oregon).

Notably however, palliative sedation was only articulated as necessarily inferior to assisted dying by interviewees in Quebec and Flanders, specifically in comparison to euthanasia. In all these cases, it was described as worse particularly from the perspective of professionals and next of kin:

Palliative sedation seems to be MAiD, only much worse; you’re put to sleep with an injection and then, well, you’ll die in a few days. What’s the point of that? You might as well just die now (. . .) (Physician 11 Quebec).

You see patients sometimes [who are] still there for many days, like four or five days, and that the family has already been saying goodbye for long time. Day three is becoming worse, day four is a day too much, day five is two days too much, and day six is really not useful for anyone anymore. And the nurses are a bit frustrated because the patient is still there, and the family is frustrated because the patient is still there, and then you sometimes think ‘Well, if this patient had asked for euthanasia, he could have passed away easily surrounded by everyone and no one would have been frustrated like this’ (Physician 5 Flanders).

Those working in palliative care described how next of kin were often wary of protracted dying, designating it as undignified, and sometimes pressuring doctors to do something to speed up the dying process. One said:

When a patient becomes unconscious, there is a malaise. People around, they say, ‘Oh my god! How long is this going to last? Doctor, can’t you do something? We don’t let a dog suffer this way, how can you let this person suffer this way?’ (Physician 16 Quebec).

Palliative sedation was described as bringing uncertainty: not knowing how long the process of unconsciousness will last, and continually having to check whether the person has died or not. One interviewee spoke of a case where they found it exhausting to be expected to talk to a sedated loved-one, who ‘surely couldn’t hear a word’. Here, assisted dying was seen as a far better option, the lawfulness of which should have made palliative sedation redundant.

### Crossing ethical and legal lines

Our interviews also revealed conceptualisations of palliative sedation that explicitly blurred the lines between alleviating symptoms and causing death: ‘You know, the limit between palliative sedation and mercy killing is a thin line sometimes’ (Physician 2 Flanders). Palliative sedation was described as more technically difficult than assisted dying, yet because it entails less bureaucracy, concerns were expressed that it would become clinically convenient. This was considered especially risky for physicians operating on their own, such as general practitioners/family physicians, and physicians in long-term treatment facilities. Whereas assisted dying was perceived as patient-driven, this was not the case with palliative sedation. Not everyone considered this to be a problem, however. One interviewee revealed having been concerned when assisted dying legislation passed, because they worried that enforcement of the law might mean that they would:

no longer dare to sedate, to help paternalistically push people over the edge, who are suffering, who hadn’t asked for euthanasia, but whom we thought it would be beneficial and helping them (Physician 2 Flanders).

Particularly in Flanders, it seems that the techniques and language of palliative sedation are sometimes used in non-requested, and therefore unlawful, ending of life. One interviewee gave examples from a nursing home and general/family practice:

They know the patients already; the patient is in a stage of dementia and the family is saying to the physician ‘well this is really what mother doesn’t want. You know how mother was (. . .) she would have [preferred to be] dead, so you have to do something, please. Help us to stop this’. And then (. . .) without thinking very well about what they are doing, they start palliative sedation and they don’t do it in a good way. (. . .) You see that more often even with general physicians. (. . .) I recently [saw] a lady who told me frankly that her physician had [performed] euthanasia on her husband who had dementia and I asked, ‘did he ask for it himself?’ and she said no (Physician 5 Flanders).

## Discussion

The different and sometimes diverging opinions and experiences with palliative sedation uncovered in our study illustrate the varied facets and non-standardised character of the practice, both between and within the three jurisdictions. Interviewees’ accounts echo concerns from the research literature: patient preferences regarding circumstances of dying; physicians’ intentions and next of kin’s experiences.

Our interviews demonstrate that palliative sedation is sometimes seen as belonging within the sphere of assisted dying, however likely more so in Quebec and Flanders than in Oregon. We believe to a significant degree this has to do with assisted dying in Oregon being self-administered and occurring mostly without health providers’ involvement beyond writing the lethal prescription. By contrast, clinicians’ involvement with assisted dying in Quebec and Flanders is ‘hands-on’ and continues until death.

For most interviewees, palliative sedation and assisted dying are perceived either as alternative interventions to be chosen depending on the situation or on the personal preferences of the patient, or as variants on the same theme. The choice of either intervention is, of course, framed by the patient’s clinician and the information they supply. We also find that some use palliative sedation explicitly to bring about death by overdosing. For others, even if death is not perceived to be hastened in palliative sedation, a consequentialist line of thought places the two interventions side-by-side: when the end result is the same, differences are reduced to ones of degree rather than of essence. Emerging from this complex and arguably muddy terrain are indications of underlying medico-cultural issues to which we now turn.

Contemporary understandings of a good death emphasise freedom from pain and unbearable symptoms of various sorts. Indeed, suffering at end of life is increasingly understood as meaningless and as disruptive to the person (Richards and Krawczyk, 2019). In different ways, both palliative sedation and assisted dying target suffering: alleviation of suffering is the one and only stated intention of palliative sedation and assisted dying advocacy increasingly relies on a discourse of suffering and pain (Hendry et al., 2012; Karsoho et al., 2016). Suffering also figures in the laws regulating assisted dying in Flanders and Quebec. Some interviewees believed that what most people *really* wanted was not to suffer, and they put many requests for assisted dying down to a lack of information about palliative care’s role in preventing their suffering. This is in keeping perhaps with the philosophy that assisted dying is redundant if good palliative care is provided.

Through the permanent alleviation of suffering and the production of stillness (through unconsciousness) achieved in palliative sedation and the termination of suffering through the production of death in assisted dying, both appear as credible options to bring about a good death. We also observe that suffering plays a key role in our interviewees’ understanding of both procedures not only as each might present a remedy for it, but also in how they might serve to exacerbate it. Specifically, we find this relates to the themes of time and control.

Preparation is considered a central tenet of the good death cross-culturally (Bloch and Parry, 1982). Intentionally creating or identifying a dying period marks an important liminal phase between life and death and may facilitate a sense of preparation. Simultaneously, Western European and North American cultures exhibit a ‘denial of dying’, whereby the notion of a discrete period of bodily decline where death is imminent is increasingly viewed as unacceptable, and assisted dying laws can be seen as a formal manifestation and legitimation of the desire to shorten this phase of life (Richards and Krawczyk, 2019). Indeed, assisted dying offers unique opportunities for choreographing death (Buchbinder, 2018) and possibly for creating a liminal phase of ‘dying’ without the patient being in a state of extreme physical precariousness. Arguably, palliative sedation too can be viewed through this lens. The practice of organising family farewells prior to rapidly induced unconsciousness in particular serves to evidence this (Bruinsma et al., 2014).

It has been observed that a permanent state of unconsciousness not only prevents further expressions of decline but also constitutes the closing or concluding of a patient’s social life (Materstvedt and Bosshard, 2009). For this reason, some have equated it to ‘social death’ (De Graeff and Dean, 2007). A straightforward interpretation of unconsciousness as social death is that it relies on a specific Cartesian intellectual heritage of mind-body dualism. Although a well-established feature of Western culture and biomedicine (Leder, 1984), there is clinical research which directly challenges the notion that the social death of unconscious persons is a necessary or incontrovertible fact (Bird-David and Israeli, 2010; Kaufman, 2000). Moreover, this line of thought does not fit well with the recurring theme, both in our interviews and in the literature, wherein a period of lingering unconsciousness is experienced either as positive and comforting, or as emotionally draining. Indeed, in either case, the patient is not considered dead. As such, palliative sedation is a preferable option for some, for the very same reason that others denounce it: namely the possibility of a gradual dying process. This may be perceived as comforting because it mimics natural dying (Seymour, 1999; Timmermans, 2005). Differently put, people may find in palliative sedation that liminal period between life and death – albeit one that is experienced only by those around the dying person, rather than by the person themselves. More commonly in our findings though, the opposite seems to be the case and lingering unconsciousness without prospect of return is presented as undignified per se.

Both in our interviews and in the reviewed literature (Claessens et al., 2011; Van Tol et al., 2015), family members are presented as responding impatiently to a protracted dying process. Kaufman’s (2005) ethnographic account of dying in American hospitals makes the point well: there is no more ‘empty time’ in hospitals today, time spent simply *waiting for death*. All time must be filled with *doing*. In the same vein, some have argued that the integration of deep sedation until death in palliative care can be seen as a move away from the discipline’s original ethos, toward one of active therapy and intervention (Twycross, 2019). In our interviews, we see that time is sometimes experienced as a nuisance which adds nothing of value. The passage of time seems to exacerbate or even cause suffering. Part of the difficulty, it appears, relates to unpredictability and hence to a lack of control. This factors heavily in the opinions of our interviewees, not least of those who perceive palliative sedation to be an inferior version of assisted dying. The issue of control is two-pronged: one relating to patients, another to professionals.

It has been argued that the (bio)medical endeavour in general stems from the Enlightenment era desire to control nature – both external and within ourselves (Komesaroff, 2008). In that light, both prolonging life and hastening death emerge as manifestations of the same medical moral imperative to act; to *do something* (Lavi, 2005). We find that both palliative sedation and assisted dying come across as imbued with notions of control. For some of our interviewees, palliative sedation was a paternalistic, clinician-driven procedure, which was sometimes employed at a doctor’s discretion, occasionally with what seems like an explicit intention to cause death. This picture is underpinned when interviewees point to practices where physicians operate alone, initiate sedation without due reflection or process, or where palliative sedation appears to be employed to mask illegal, drug-induced ending of life. Reports that physicians sometimes redirect requests for assisted dying toward palliative sedation indicate a high measure of control. In this light, we might also understand the discomfort – even suffering – associated with protracted dying as indicating powerlessness, and the push to increase dosages to hasten death as a desire to regain control.

Contrary to this, assisted dying emerges in our interviews as a patient-driven practice. Indeed, it is often championed as an approach favouring self-determination and authorship over one’s own life. Throughout the interviews, patients requesting assisted dying were described in strikingly similar terms and were often seen as having a particular character. Their desire for assisted dying was taken to reflect who they were as a person rather than being a reaction to their condition, which is also supported in the literature (Richards, 2017; Selby et al., 2019). Thus, where palliative sedation becomes the option for patients who want to ‘live through their disease’ until it might become unbearable, what interviewees suggest is that this is not the main motivation of many patients requesting assisted dying. This indicates a belief that palliative sedation cannot be a substitute for assisted dying as the latter serves a different purpose than to produce a good death understood narrowly as one free of patient-experienced suffering.

## Limitations

The study has some limitations. We used purposive sampling and snowballing to recruit our sample and it is not a representative selection of interviewees. A study employing a more randomized strategy of recruitment might produce somewhat different findings. Similarly, studying the perceptions of lay persons might have yielded different results, which would be valuable to a holistic understanding of the topic. This exceeded the scope of this study but remains an objective for future research.

The specific importance of palliative sedation as a palliative treatment was unforeseen to us when designing the study. We accordingly did not formulate specific questions about the relationship between palliative sedation and assisted dying, but rather asked follow-up questions when interviewees raised the issue. Asking each interviewee directly about their views on palliative sedation might have garnered even more insights into the topic. Nevertheless, the organic way in which interviewees commented on the topic indicates that it is perceived as having specific relevance to assisted dying. We therefore believe it is valuable to focus on this topic which emerged from the data in an inductive way.

## Concluding remarks

Relief of suffering is central to end of life care and emerges as a key concept in both palliative sedation and assisted dying, and both interventions may be seen to target and also to exacerbate suffering. In particular, some interviewees take issue with palliative sedation, arguing that it aggravates suffering for those around the patient for reasons to do with lingering, lack of control and the passing of time. We observe a prominent way of thinking in which *unproductive* time is seen as useless. Assisted dying affords the possibility of creating a liminal space actively chosen by patients, in an effort to take control. It can produce death in a way that avoids both lingering and the last stages of bodily decline. In the case of those for whom the gradual dying afforded by palliative sedation is positively regarded, this may be precisely because they see utility in this dying time, as a liminal phase.

Control is integral to the practice of medicine and to the critique of medicalisation. In different ways, both palliative sedation and assisted dying appear saturated with issues of control. For some in our study, palliative sedation had an aura of paternalism, used by physicians at their discretion and sometimes deliberately employed to ‘help’ patients die faster. On the other hand, for some interviewees assisted dying was portrayed as an intervention which put patients in control. A key point of distinction was that requests for palliative sedation were understood to stem from a desire not to *suffer*, whereas requests for assisted dying were about a desire to *control*. As such, the choice between the two relates not so much to illness conditions or symptoms, but to a person’s character.

Our findings show that in the opinion of several of those interviewed, both palliative sedation and assisted dying can work to produce a ‘good death’. Crucially, however, it does not appear that assisted dying legislation renders palliative sedation redundant in the minds of everybody involved in end of life care, nor do they see palliative sedation as an alternative intervention that makes assisted dying unnecessary. Moreover, the categorical distinction between palliative sedation and assisted dying which has been much attended to by expert groups and the creators of definitions and guidelines, is not always so categorically distinct in real life clinical encounters – a reality that warrants further clinical and social science research.
